# Caffeine in Opium—Coma

**Published:** 1861-09

**Authors:** 


*Caffeine* (*in Opium*)—*Coma.* Dr. H. F. Campbell reports a case of poisoning by opium, treated with Caffeine. A man, aged about 28 years, had taken nearly three ounces of laudanum. Fifteen hours later he was found entirely unconscious, face of a dark purple hue; hands and feet also purple from congestion, nails on fingers and toes of an indigo color, patches of venous congestion all over the surface; respiration not quite four to the minute, in spite of constant slapping and shaking, and apparently greatly obstructed by the accumulation of mucous; pulse very feeble, about 100; muscular system completely relaxed, so that the head fell about by its own weight, and arms and legs obeyed only the influence of gravity. Ice was applied to the scalp, ice-water poured from a distance to the head, and about twenty-five grains of caffeine, dissolved in two ounces of cold water, introduced into the rectum by means of a small syringe. In thirty-five minutes the respiration seemed less difficult and more regular, eight to the minute; the skin began to present less of the cerulean tint. In one hour after, the respiration had risen to twelve, and shortly rose to sixteen in the minute, when the skin was nearly of the natural hue, though the nails on both hands and feet still remained of a purplish cast. Slight spasmodic movements in the fingers were now observed, and also some occasional subsultus in the muscles of the fore-arm; the under-lip, which before was hanging, now became elevated and slightly compressed against the teeth. When the hand of the patient was held, and an attempt made to extend the arm at the elbow, decided muscular resistance was observed. The lid of the left eye was noticed to be raised and let down rapidly once or twice. The pulse had now become full and somewhat resisting, and the action of the heart tumultuous. On being raised, the patient once mode a noise slightly resembling a groan, but never manifested the least consciousness. For a short time the mucous rale in the chest seemed somewhat to diminish; later, however, it became more and more obstructive, the gurgling reaching up into the throat, and threatening to strangle the patient. On turning him upon the right side, a bloody mucous bubbled out of the nostrils. Number of respirations twenty to the minute; surface of the body entirely hot. The ice was constantly kept on the head, and mustard plasters applied to the spine as well as to the extremeties. Death ensued, apparently from the accumulation of bloody mucus in the bronchial tubes and larynx, in consequence of the long enduring pulmonary congestion existing previous to the administration of the caffeine. Instead of proving anything against the antidote, the surprising effect of it in this case rather confirms its value.—*Southern Medical and Surgical Journal.*

